# *Candida albicans* Genes Modulating Echinocandin Susceptibility of Caspofungin-Adapted Mutants Are Constitutively Expressed in Clinical Isolates with Intermediate or Full Resistance to Echinocandins

**DOI:** 10.3390/jof10030224

**Published:** 2024-03-19

**Authors:** Anshuman Yadav, Sudisht K. Sah, David S. Perlin, Elena Rustchenko

**Affiliations:** 1 Department of Biochemistry and Biophysics, University of Rochester Medical Center, Rochester, NY 14642, USA; anshuman@urmc.rochester.edu (A.Y.); sudisht_sah@urmc.rochester.edu (S.K.S.); 2 Hackensack Meridian School of Medicine, Center for Discovery and Innovation, Nutley, NJ 07110, USA; david.perlin@hmh-cdi.org

**Keywords:** *Candida albicans*, echinocandin susceptibility, drug adaptation, clinical isolates

## Abstract

The opportunistic fungus *Candida albicans* is the leading cause of invasive candidiasis in immune-compromised individuals. Drugs from the echinocandin (ECN) class, including caspofungin, are used as a first line of therapy against invasive candidiasis. The only known mechanism of clinical resistance to ECNs is point mutations in the *FKS1* gene, which encodes the drug target. However, many clinical isolates developed decreased ECN susceptibilities in the absence of resistance-associated *FKS1* mutations. We have identified 15 *C. albicans* genes that contribute to decreased drug susceptibility. We explored the expression of these 15 genes in clinical isolates with different levels of ECN susceptibility. We found that these 15 genes are expressed in clinical isolates with or without *FKS1* mutations, including those strains that are less susceptible to ECNs. In addition, *FKS1* expression was increased in such less susceptible isolates compared to highly susceptible isolates. Similarities of gene expression patterns between isolates with decreased ECN susceptibilities in the absence of *FKS1* mutations and clinically resistant isolates with mutations in *FKS1* suggest that clinical isolates with decreased ECN susceptibilities may be a precursor to development of resistance.

## 1. Introduction

Invasive fungal diseases, including those caused by *Candida* species, are rising overall, particularly among immunocompromised individuals. Indeed, *C. albicans*, a predominant fungal pathogen of humans, is the most common cause of systemic candidiasis. Along with infections by non-*albicans* species, *C. albicans* increasingly displays resistance to antifungals [1,2,3,4,5,6].

Drugs from the echinocandin (ECN) class caspofungin, anidulafungin, micafungin, and rezafungin, are recommended as a first-line treatment for invasive candidiasis because of their low toxicity and high efficacy, especially against isolates that are resistant to azole drugs [7,8]. ECNs inhibit the fungal-specific target 1,3-β-glucan synthase, an enzyme complex required for the synthesis of glucan, the main structural skeleton of the cell wall to which all other components are cross-linked [9,10,11].

Currently, there is only one known mechanism of *C. albicans* clinical resistance to ECNs. This involves point mutations in the *FKS1* (orf19.2929) gene, which encodes a catalytic subunit of the 1,3-β-glucan synthase complex, in various *Candida* species and in the *FKS1* gene or in the orthologous *FKS2* gene in *C. glabrata* [12,13]. These mutations decrease the sensitivity of glucan synthase to ECNs by several orders of magnitude, dramatically elevate minimum inhibitory concentrations (MICs), and confer reduced pharmacodynamic responses [12,14,15,16]. The genome of *C. albicans* also contains two orthologous *FKS* genes, *FKS2* (orf19.3269) and *FKS3* (orf19.2495), which are conserved in other *Candida* species and in other microbes. We have recently shown that these genes are negative regulators of *FKS1* [17].

Importantly, work from our lab and others have established that *C. albicans* possesses mechanisms independent of *FKS1* mutations that confer decreased susceptibility to ECNs, although these mechanisms do not confer clinical resistance [18]. Indeed, dozens of *Candida* clinical isolates have been identified that display a wide range of increased MIC values for ECNs, including some at or below the MIC breakpoints but lacking classical *FKS1* resistance mutations [19,20,21]. Mechanisms that decrease drug susceptibility in the absence of *FKS1* mutations are considered to be of critical importance as they allow transient survival in the presence of cidal ECNs and facilitate the evolution of classical clinical resistance [22,23].

We have previously used *C. albicans* caspofungin-adapted mutant modeling clinical isolates with decreased ECN susceptibility in the absence of *FKS1* mutation to identify genes that are involved in the control of ECN susceptibility. We have identified a total of 15 genes, 10 of them on Ch5 and 5 of them on Ch2, that are simultaneously downregulated (Ch5 genes) or upregulated (Ch2 genes) in the mutants that adapted to growth in the presence of otherwise lethal amounts of ECN caspofungin [18]. These genes control the amounts of three major cell wall components—glucan, mannan, and chitin—as well as masking/unmasking of immunological epitope glucan [18]. Here, we set about to address the expression of these 15 genes in various clinical isolates that differ by their level of susceptibility to drugs from the ECN class.

## 2. Materials and Methods

### 2.1. Genes, Clinical Isolates, and Primers

*C. albicans* genes and clinical isolates studied here are presented in Table 1 and Table 2 correspondingly. Primers used here are presented in Table S1.

### 2.2. Maintenance and Growth of Strains and Media

Cells were maintained, stored, and grown using our standardized approach that prevents the induction of chromosome instability, as previously described [24]. This approach favors maintaining a population of cells that represents a major fraction of cells [25]. Cells were stored in a 25% (vol/vol) glycerol solution at −80 °C to interrupt metabolism and routinely grown at 37 °C. When needed, cells from a −80 °C stock were streaked for independent colonies onto yeast extract–peptone–dextrose (YPD) plates and incubated at 37 °C until young colonies with a size of approximately 1 × 10^5^ to 3 × 10^5^ cells/colony grew up. Young colonies were collected, a proper dilution in sterile water was prepared with the aid of a hemacytometer, approximately 3000 colony forming units (CFU) were plated onto each plate, and plates were incubated until young colonies appeared. 

We used YPD medium (1% yeast extract, 2% peptone, and 2% dextrose) and RPMI 1640 medium (Sigma, St. Louis, MO, USA). To prepare solid medium, 2% (wt/vol) agar was added.

### 2.3. Broth Microdilution Assay to Determine MICs

We employed a broth microdilution assay in accordance with the CLSI document M27-A3 broth microdilution method for yeasts [26]. The assay was performed in triplicate. *C. parapsilosis* ATCC 22019 and *C. krusei* ATCC 6258 were used as quality control strains.

### 2.4. Determination of Gene Expression by qPCR

The clump of cells of the clinical isolate was removed from −80 °C frozen stock and suspended in a 50 mL culture tube containing 10 mL YPD medium (primary culture). The culture tube was incubated in a shaker at 37 °C for 16 h at 220 rotations per minute (rpm). A fresh 10 mL YPD medium in a 50 mL culture tube was inoculated with 2% saturated primary culture and incubated in a shaker at 37 °C for 4.5 h at 220 rpm. Cells were collected and washed twice with ice-cold water to remove any residual medium. The cells were suspended in 0.6 mL lysis buffer provided in an RNeasy kit (Qiagen, Germantown, MD, USA) and transferred in a 1.5 mL tube containing 200 µL of glass beads (0.5 mm). The cells were broken by 6 alternative cycles of 1 min for each vortex and sample incubation on ice. The cell lysate was centrifuged at 10,000 rpm for 3 min at 4 °C, the supernatant was collected in a new 1.5 mL tube, and total RNA was extracted according to RNeasy kit instruction. A total of 2 μg of total RNA was treated with DNase I (Thermo Fisher Scientific, Waltham, MA, USA) followed by cDNA preparation using multiscript reverse transcriptase (Applied Biosystems, Bedford, MA, USA) as per manufacturer’s instruction. The quantitative amplification of cDNA was monitored by incorporation of SYBR green in the StepOnePlus qPCR system (Applied Biosystems, MA, USA) using a PCR master mix from Applied Biosystems (MA, USA). The amplification curves were analyzed using StepOne software v2.2.2 (Applied Biosystems, Bedford, MA, USA).

We used the expression of the *ACT1* (orf19.5007) gene to normalize the expression of genes of interest or target genes [27]. We used SC5314 (Candida Genome Database: http://www.candidagenome.org/cache/C_albicans_SC5314_genomeSnapshot.html, accessed on 9 October 2023) as a control strain. MICs for caspofungin, anidulafungin, or micafungin of SC5314 are, correspondingly, 0.06 mg/L, 0.06 mg/L, and 0.015 mg/L [28]. We used the ΔΔCt method for the relative quantification of mRNA of target genes [29]. Briefly, the expression ratio of clinical isolate/control strain = 2^−∆∆Ct^

where

∆∆Ct = [∆Ct clinical isolate − ∆Ct control strain];

∆Ct clinical isolate = Ct_target gene_ − Ct*_ACT1_*;

∆Ct control strain = Ct_target gene_ − Ct*_ACT1_*;

Ct = threshold cycle value.

The quantification and statistical analysis (Student’s *t*-test) was performed in Excel version 16.0 (Microsoft 365). Graphs were made using GraphPad Prism (9.5.0).

## 3. Results and Discussion

As presented in the Introduction, we employed model caspofungin-adapted mutants in which we identified 10 downregulated genes on Ch5 and 5 upregulated genes on Ch2 (Table 1) that act in concert to modulate drug-susceptibility. We set about to address how these 15 genes are expressed in clinical isolates. We used a qPCR approach (Section 2) to analyze the expression of the above genes in 11 *C. albicans* clinical isolates representing three levels of ECN MIC-based in vitro susceptibility. The levels corresponded to highly ECN-susceptible isolates, less susceptible isolates, and clinically resistant isolates (Table 2). Clinically resistant isolates possessed different *FKS1* mutations to resistance in contrast to the other mutation-free isolates (Table 2). A total of five highly susceptible isolates, DPL253, DPL255, DPL258, DPL263, and DPL266, have MIC values for ECNs caspofungin, anidulafungin, or micafungin, ranging from 0.015 to 0.06 mg/L (Table 2). A total of three less susceptible isolates, DPL225, DPL291, and DPL1000, have MIC values ranging from 0.05 to 0.40 mg/L (Table 2). Finally, a total of three clinically resistant isolates, DPL15, DPL1009, and DPL1008, have relatively high MIC values ranging from 0.5 to 8.0 mg/L (Table 2).

In addition to 15 genes that control cell wall remodeling, we determined the expression of *FKS* genes that are responsible for the biosynthesis of cell wall constituents (Table 1, see Section 1). In earlier studies addressing the relation between expression of *C. albicans FKS* genes and susceptibility to ECNs, ref. [14] found changes in relative expression ratios of *FKS1*/*FKS2* and *FKS1*/*FKS3* in various clinical isolates, whereas [30] described expression changes in *FKS* genes in caspofungin-adapted model mutants vs. their drug-susceptible parentals. More recently, ref. [18] showed that 10 Ch5 genes that we are analyzing here control expression changes in *FKS* genes. By including *FKS* genes in this work, we addressed the question of how expressions of *FKS*s relate to the expression of 15 genes on Ch2 and Ch5 in clinical isolates.

In order to compare gene expressions from different isolates, we used a control *C. albicans* strain SC5314, which is the reference strain for sequencing (see Section 2). Caspofungin, anidulafungin, and micafungin MICs of SC5314 are, correspondingly, 0.06 mg/L, 0.06 mg/L, and 0.015 mg/L [28]. Each of the 15 Ch2 and Ch5 genes was compared to the corresponding genes in SC5314 (Section 2). In a comparison of expression changes across all isolates, we found that any Ch2 or Ch5 gene may vary. In several isolates, we observed changes in concert with either all 5 Ch2 genes or all 10 Ch5 genes.

**Figure 1:**
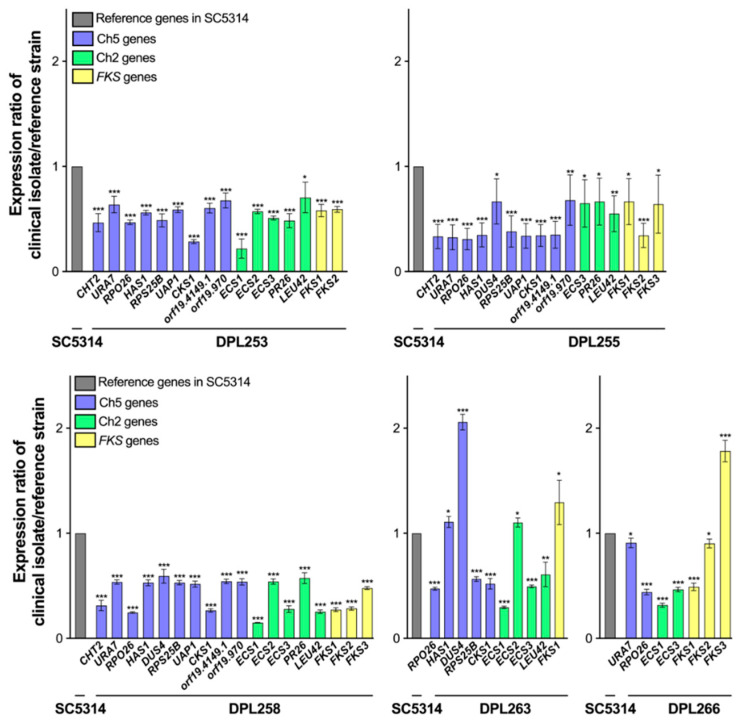
Patterns of relative gene expression in clinical isolates DPL253, DPL255, DPL258, DPL263, and DPL266 that are highly susceptible to echinocandins (see Table 2). Gene expressions were measured with qPCR, normalized against *ACT1,* and compared to the corresponding genes of the reference strain SC5314, in which expressions were considered 100%. Shown is the average of three independent experiments ± standard deviations. Only genes with significant changes are presented as bars. See Supplementary Materials for bar graphs, including both significant and nonsignificant data. The asterisks indicate a *p* value of <0.05 (*), <0.01 (**), or <0.001 (***), as determined using Student’s *t*-test. The graph was prepared using GraphPad Prism (9.5.0).

**Figure 2:**
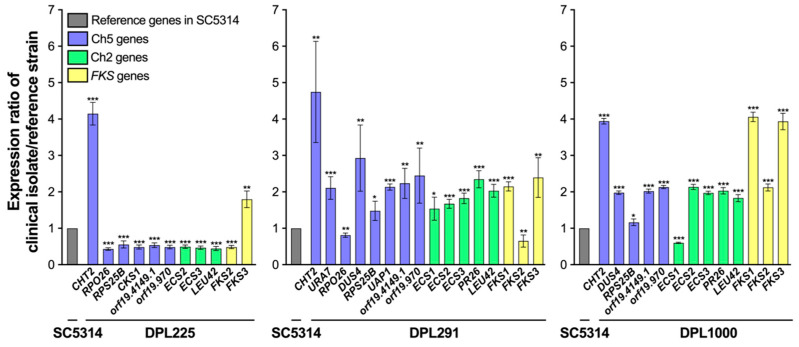
Patterns of relative gene expression in clinical isolates DPL225, DPL291, and DPL1000 that are less susceptible to echinocandins (see Table 2). The asterisks indicate a *p* value of <0.05 (*), <0.01 (**), or <0.001 (***), as determined using Student’s *t*-test. The graph was prepared using GraphPad Prism (9.5.0). For more details, see legend of Figure 1.

**Figure 3:**
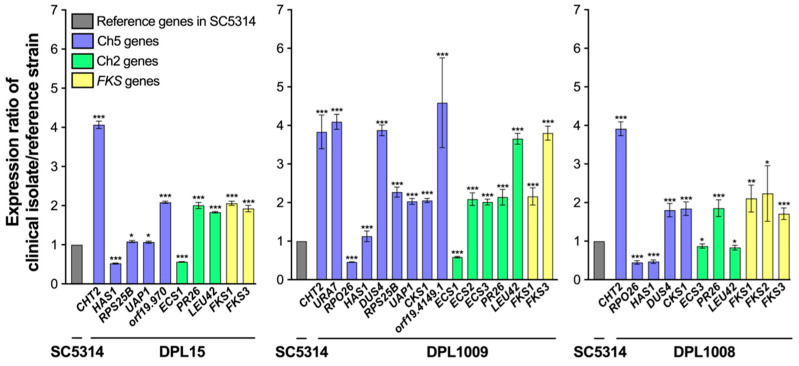
Patterns of relative gene expression in clinically resistant isolates DPL15, DPL1009, and DPL1008 (see Table 2). The asterisks indicate a *p* value of <0.05 (*), <0.01 (**), or <0.001 (***), as determined using Student’s *t*-test. The graph was prepared using GraphPad Prism (9.5.0). For more details, see legend of Figure 1.

Strikingly, we found that expression patterns of highly susceptible isolates (Figure 1) showed more similarity. Also, there was more similarity of expression patterns within two other groups: isolates with decreased susceptibility and clinical resistant isolates (Figure 2 and Figure 3). Relative gene expression in highly susceptible isolates, including clinically important *FKS1*, is predominantly decreased or, in few instances, the same or higher, as compared to corresponding genes in the control strain (Figure 1). In contrast, relative gene expression in less susceptible isolates and in clinically resistant isolates are either decreased or increased (Figure 2 and Figure 3). Most importantly, in contrast to highly susceptible isolates, these patterns have a prominent feature of upregulation of *CHT2* (org19.3895) and *FKS1*. *CHT2* encodes a GPI-anchored chitinase involved in the hydrolysis of cell wall chitin, and its protein acts as a negative regulator of ECN susceptibility [18]. Interestingly, *CHT2* is also upregulated in *C. albicans* cells grown as biofilm in the presence of the bacterium *Streptococcus mutans* [31]. Also important to this study is that *CHT2* is repressed in the core caspofungin response [32,33]. Clarification of the *CHT2* role needs further study. Regarding *FKS1*, we believe that its upregulation contributes to cell wall remodeling and subsequently to decreased drug susceptibility [18]. Overall, two genes that control the amount of two major components of the cell wall, chitin (*CHT2*) and glucan (*FKS1*), are overexpressed in strains with less susceptible genetic backgrounds in striking contrast with highly susceptible isolates. Overall, the gene expression pattern of isolates with less susceptible resembles the patterns observed with *FKS*-mediated resistant isolates (Figure 2 and Figure 3).

In conclusion, there is a substantial difference between the genetic backgrounds of highly ECN drug-susceptible isolates and isolates with decreased ECN susceptibility, as well as clinically resistant isolates. All 15 genes on Ch2 and Ch5 that contribute to modulating ECN drug susceptibility are actively regulated in isolates with less ECN susceptible and in clinically resistant isolates. Our data further the narrative that isolates with less ECN susceptible and no FKS1 mutation likely contribute to the evolution of clinically resistant isolates with mutation to resistance in FKS1. Finally, our data validate our caspofungin-adapted model mutants by showing that genes identified in the in vitro model system have relevance in clinical isolates.

## Figures and Tables

**Table 1:** List of genes analyzed in this study *.

Standard Name	Assembly 19/21 Identifier
**Genes on chromosome 2**	
*ECS1*	orf19.1766
*ECS2*	orf19.6867
*ECS3*	orf19.5833
*PR26*	orf19.5793
*LEU42*	orf19.1375
**Genes on chromosome 5**	
*CHT2*	orf19.3895
*URA7*	orf19.3941
*RPO26*	orf19.2643
*HAS1*	orf19.3962
*DUS4*	orf19.966
*RPS25B*	orf19.6663
*UAP1*	orf19.4265
*CKS1*	orf19.1282
	orf19.4149.1
	orf19.970
***FKS* genes**	
*FKS1*	orf19.2929
*FKS2*	orf19.3269
*FKS3*	orf19.2495

* For more information about Ch2 and Ch5 genes, see [18]. For more information about *FKS* genes, see [17].

**Table 2:** List of clinical isolates used in this study. Also shown are MIC values of three ECNs: caspofungin (CAS), micafungin (MFG), and anidulafungin (ANI).

Strain	*FKS1* Mutation to Resistance	MIC (mg/L)		
		CAS	MFG	ANI
**Highly ECN susceptible isolates (low MIC values)**				
DPL253	none	<0.06	<0.06	<0.03
DPL255	Same as above	<0.03	<0.03	<0.03
DPL258	Same as above	0.03	0.015	0.03
DPL263	Same as above	<0.06	<0.06	<0.03
DPL266	Same as above	0.015	0.015	0.015
**Less ECN susceptible isolates (elevated MIC values)**				
DPL225	Same as above	0.12	0.12	0.12
DPL291	Same as above	0.1	0.1	0.08
DPL1000	Same as above	0.40	0.05	0.08
**Clinically resistant isolates (high MIC values)**				
DPL15	F641S	4.0	0.5	1.0
DPL1009	S645Y	4.0	4.0	2.0
DPL1008	S645P	8.0	4.0	1.26

## Data Availability

Data are contained within the article and Supplementary Materials.

## Supplementary Materials

The following supporting information can be downloaded at: https://www.mdpi.com/article/10.3390/jof10030224/s1. Table S1: List of primers used in qPCR. Figure S1: Patterns of relative gene expression in clinical isolates DPL253, DPL255, DPL258, DPL263 and DPL266 that are highly susceptible to echinocandins (see Table 2). Gene expressions were measured with qPCR, normalized against *ACT1* and compared to the corresponding genes of the reference strain SC5314 in which expressions were considered 100%. Shown is the average of three independent experiments ± standard deviations. Note that *FKS3* was not detected in DPL263. The asterisks indicate *p* value of <0.05 (*), <0.01 (**) or <0.001 (***), as determined using Student’s *t* test. The graph was prepared using GraphPad Prism (9.5.0). Figure S2: Patterns of relative gene expression in clinical isolates DPL225, DPL291 and DPL1000 that are less susceptible to echinocandins (see Table 2). The asterisks indicate *p* value of <0.05 (*), <0.01 (**) or <0.001 (***), as determined using Student’s *t* test. The graph was prepared using GraphPad Prism (9.5.0). For more details, see legend of Figure S1. Figure S3: Patterns of relative gene expression in clinically resistant isolates DPL15, DPL1009 and DPL1008 (see Table 2). The asterisks indicate *p* value of <0.05 (*), <0.01 (**) or <0.001 (***), as determined using Student’s *t* test. The graph was prepared using GraphPad Prism (9.5.0). For more details, see legend of Figure S1.
