# Supplementary material for: Candida albicans Genes Modulating Echinocandin Susceptibility of Caspofungin-Adapted Mutants Are Constitutively Expressed in Clinical Isolates with Intermediate or Full Resistance to Echinocandins
Source: J Fungi (Basel). 2024 Mar 19;10(3):224. doi: 10.3390/jof10030224 (PMC10971431; doi:10.3390/jof10030224)
Supplement: Supplementary file 1 [file jof-10-00224-s001.zip › Table S1 and supplemental figure legend.pdf]

Supplemental Material

Table S1. List of primers used in qPCR

**Target gene**	**Primer name and sequence**
CHT2 (orf19.3895)	CaCHT2 F, GTTACCCAATCTACCACCACTAC<br>CaCHT2 R, GGAGGAAGATGAAGTGACAATCT
URA7 (orf19.3941)	CaURA7 F, AACTGGGAAGAGAATGGAGATG<br>CaURA7 F, CAAGGAATGGTCGTGATGGA
RPO26 (orf19.2643)	CaRPO26 F, GGTGGATTTGGACCTGATGAT<br>CaRPO26 R, TGGTCATATACGGAGTGGTAGT
HAS1 (orf19.3962)	CaHAS1 F, AGCCAAGGGTGTGAACTTATTA<br>CaHAS1 R, CGGCTTCATCAATCACCAAAG
DUS4 (orf19.966)	CaDUS4 F, CTGCCAAAGACAAGTATGGAGA<br>CaDUS4 R, CGCTTCCACCACCTTATTGA
RPS25B (orf19.6663)	CaRPS25B F, GGTGGTTCTTTAGCCAGAGTT<br>CaRPS25B R, GCACGAGTGTAGATAGCTTGTT
UAP1 (orf19.4265)	CaUAP1 F, ACCTAATGAACAAACAGCATCAAC<br>CaUAP1 R, CTTGACCTCCTGCCATCAATAA

CKS1 (orf19.1282)	CaCKS1 F, CAGAAGAAGAATGGAGAGGGTTAG<br><br>CaCKS1 R, CCGGGATTTAAGGGTCTCTTG
orf19.4149.1	Ca19.4149.1 F, TCCAGCTTTCAGAGTTGAAGAA<br><br>Ca19.4149.1 R, TCTAGAAGCACCAGCAGAAAC
orf19.970	Ca19.970 F, TATCAGGTGCTTGGCTTTGG<br><br>Ca19.970 R, GGAAACCTCGGCATCAAGTAA
ECS1 (orf19.1766)	CaECS1 F, GCAGTTGCTGGTAAAGTCATTG<br><br>CaECS1 R, GGCAAACCAACCTTGAGAATTT
ECS2 (orf19.6867)	CaECS2 F, CTCGTTCCCAATTAGCCCAATA<br><br>CaECS2 R, AGGACCATAGCTGTTGGAATTAG
ECS3 (orf19.5833)	CaECS3 F, TGTGTGGTGGAGACAGATTTAG<br><br>CaECS3 R, ATGGGTTTGCTCAGTGGTT
PR26 (orf19.5793)	CaPR26 F, GAGGGCACAAGAAGAAGTCAA<br><br>CaPR26 R, CCAGTGGTGGATGAAACTATACC
LEU42 (orf19.1375)	CaLEU62 F, TTGAAATGGCCACTCCTAACA<br><br>CaLEU62 R, CAGCAACACTACACCCTCTATC

FKS1 (orf19.2929)	CaFKS1 F, GGATATCAAGACCAAGCCAACTA<br>CaFKS1 R, CCAGGAGTTTGACCACCATAA
FKS2 (orf19.3269)	CaFKS2 F, CTAGCAGTCGCCAATCATGTA<br>CaFKS2 R, CCGATAATGCAAACCCAAGAAC
FKS3 (orf19.2495)	CaFKS3 F, AGCTTGGTGCCCTGAAA<br>CaFKS3 R, GTTGCTGACATTATCGTCTTGG
ACT1 (orf19.5007)	CaACT1 F, ACTACCATGTTCCCAGGTATTG<br>CaACT1 R, CCACCAATCCAGACAGAGTATT

Supplemental figure legend

Figure S1. Patterns of relative gene expression in clinical isolates DPL253, DPL255, DPL258, DPL263 and DPL266 that are highly susceptible to echinocandins (see Table 2). Gene expressions were measured with qPCR, normalized against *ACT1* and compared to the corresponding genes of the reference strain SC5314 in which expressions were considered 100%. Shown is the average of three independent experiments ± standard deviations. Note that *FKS3* was not detected in DPL263. The asterisks indicate *P* value of <0.05 (*), <0.01 (**) or <0.001 (***), as determined using Student's *t* test. The graph was prepared using GraphPad Prism (9.5.0).

Figure S2. Patterns of relative gene expression in clinical isolates DPL225, DPL291 and DPL1000 that are less susceptible to echinocandins (see Table 2). The asterisks indicate P value of <0.05 (*), <0.01 (**) or <0.001 (***), as determined using Student's t test. The graph was prepared using GraphPad Prism (9.5.0). For more details, see legend of Fig. S1.

Figure S3. Patterns of relative gene expression in clinically resistant isolates DPL15, DPL1009 and DPL1008 (see Table 2). The asterisks indicate P value of <0.05 (*), <0.01 (**) or <0.001 (***), as determined using Student's t test. The graph was prepared using GraphPad Prism (9.5.0). For more details, see legend of Fig. S1.
