# Supplementary figures and images for: Candida albicans Genes Modulating Echinocandin Susceptibility of Caspofungin-Adapted Mutants Are Constitutively Expressed in Clinical Isolates with Intermediate or Full Resistance to Echinocandins
Source: J Fungi (Basel). 2024 Mar 19;10(3):224. doi: 10.3390/jof10030224 (PMC10971431; doi:10.3390/jof10030224)

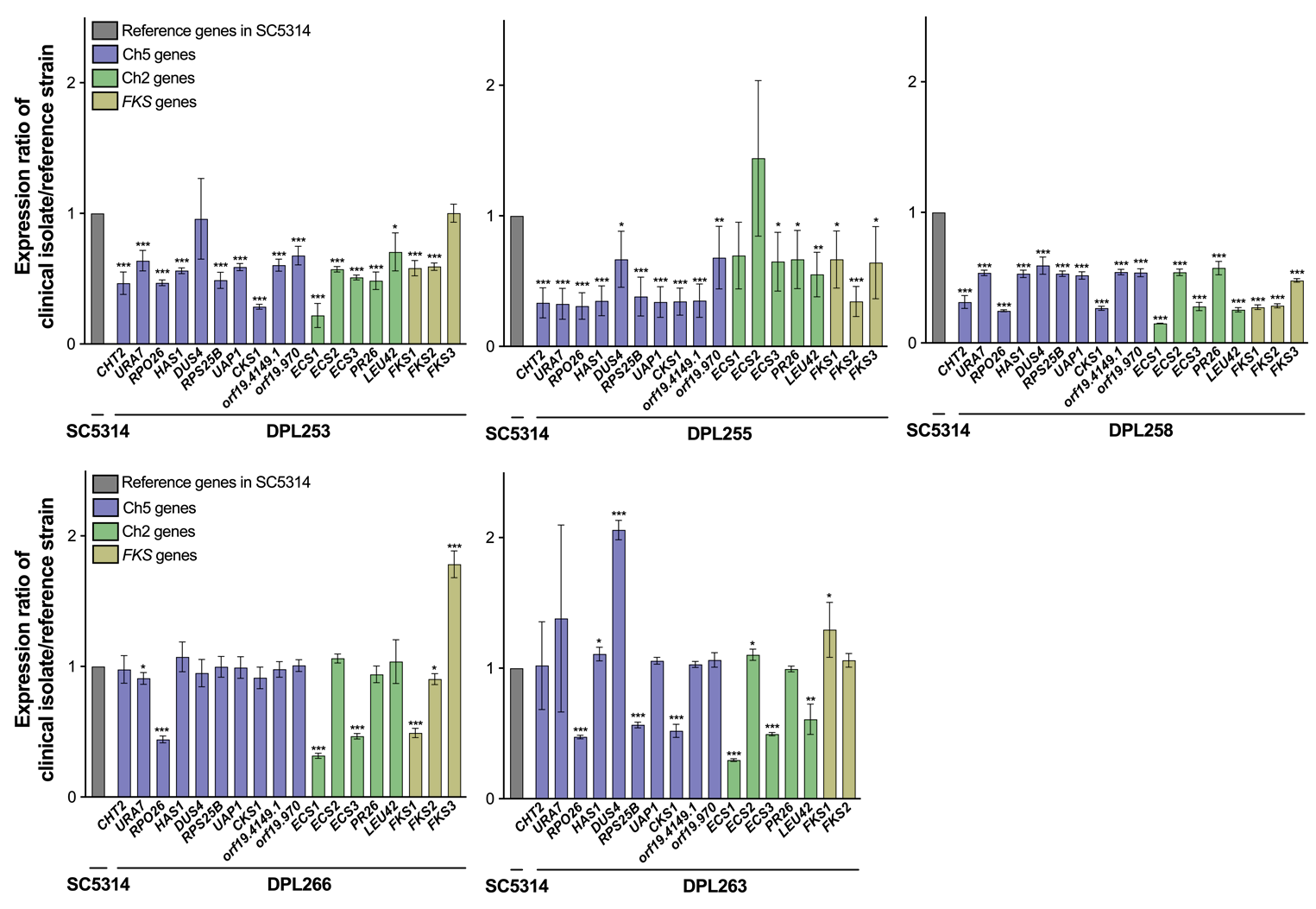

Supplement: Supplementary file 1 [file jof-10-00224-s001.zip › Figure S1.tif]

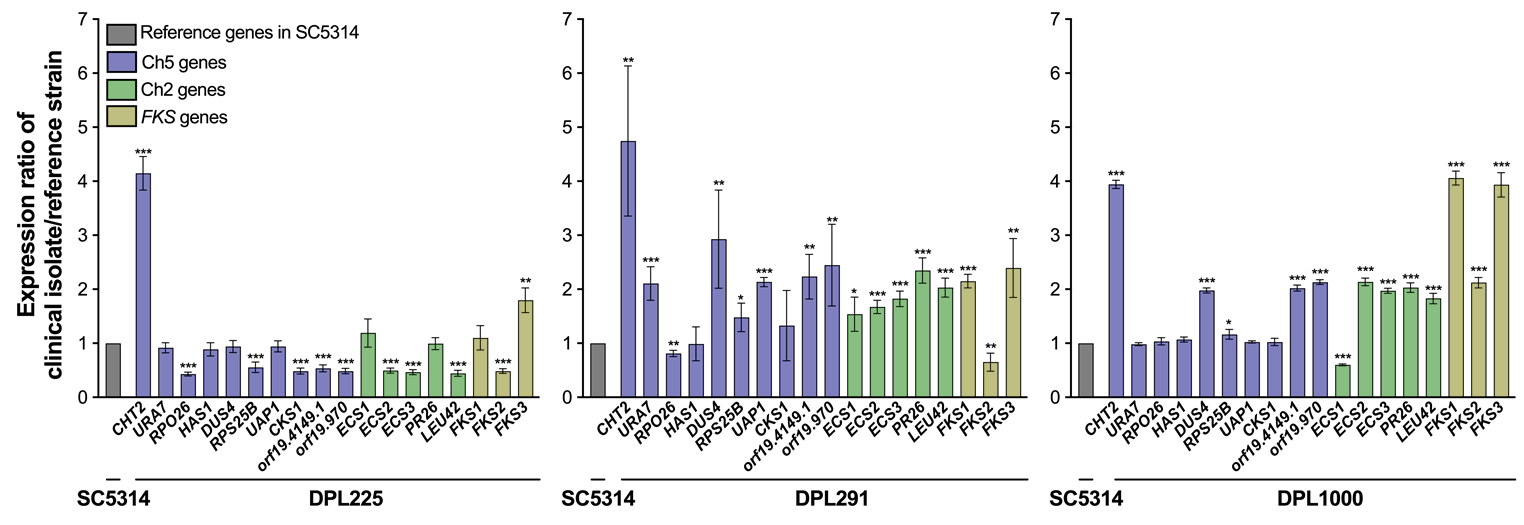

Supplement: Supplementary file 1 [file jof-10-00224-s001.zip › Figure S2.tif]

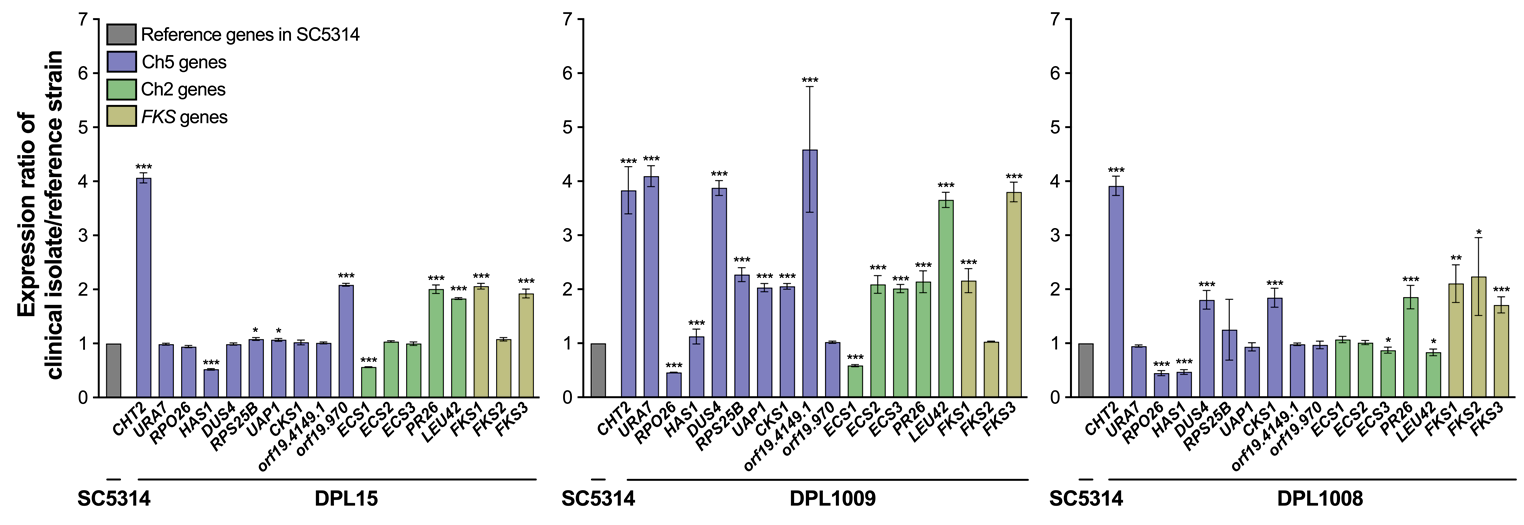

Supplement: Supplementary file 1 [file jof-10-00224-s001.zip › Figure S3.tif]
